# Low serum uric acid levels increase the risk of all-cause death and cardiovascular death in hemodialysis patients

**DOI:** 10.1080/0886022X.2020.1745234

**Published:** 2020-03-29

**Authors:** Ming Li, Zeng-Chun Ye, Can-Ming Li, Wen-Bo Zhao, Hua Tang, Xun Liu, Hui Peng, Tan-Qi Lou

**Affiliations:** Department of Nephrology, The Third Affiliated Hospital of Sun Yat-sen University, Guangzhou, People’s Republic of China

**Keywords:** Hemodialysis, serum uric acid, all-cause mortality, cardiovascular mortality

## Abstract

**Background:**

Elevated serum uric acid (SUA) is associated with increased cardiovascular (CV) and all-cause mortality risk in the general population, but the impact of UA on mortality in hemodialysis patients is still controversial. The aim of the study was to explore the relationship between SUA and all-cause mortality and CV mortality in hemodialysis patients.

**Methods:**

This retrospective, observational cohort study included 210 HD patients with a mean age of 56.6 ± 16.6 years. All demographic and laboratory data were recorded at baseline. The Kaplan–Meier method and Cox proportional hazard regression model were used to examine the association between SUA and all-cause mortality and CV mortality in HD patients.

**Results:**

With 420 µmol/L (20th percentile) and 644 µmol/L (80th percentile) as the boundary points, the patients were divided into three groups. After a median follow-up of 49.8 months, 68 (32.4%) all-cause deaths and 34 (16.2%) CV deaths were recorded. The Kaplan–Meier method showed that with a decrease in SUA, all-cause mortality (log rank *χ*^2^ = 15.61, *p* = .000), and CV mortality (log rank *χ*^2^=14.28, *p* = .000) increased. Each 100 µmol/L increase in SUA was associated with lower all-cause mortality with an hazard ratio (HR) of 0.792 (0.645–0.972) and lower CV mortality with an HR of 0.683 (0.505–0.924) after adjusting for age, sex, and complications. Compared to the lowest quartile, all-cause mortality [HR 0.351(0.132–0.934), *p* = .036] and CV mortality [HR 0.112 (0.014–0.925), *p* = .042] were lower in the highest SUA quartile.

**Conclusion:**

A lower SUA level in HD patients was associated with a higher risk of all-cause mortality and CV mortality. Moreover, higher SUA concentrations may be cardioprotective in HD patients.

## Introduction

Uric acid is the final product of purine nucleotide catabolism, and hyperuricemia is very common in the clinical setting. Hyperuricemia is closely related to hypertension, diabetes mellitus, metabolic syndrome, and cardiovascular (CV) diseases in the general population [1–3]. In addition, studies have shown that hyperuricemia is an important factor in the progression of nondialysis CKD, and higher UA values are associated with higher all-cause and CV mortality among patients with stage 3 or 4 CKD [4,5]. In ESRD patients, with a decline in renal function, the prevalence of hyperuricemia is much higher, with an incidence as high as 50% [3]. However, in hemodialysis patients, the relationship between serum uric acid (SUA) and prognosis is much more complex than that of nondialysis CKD patients. Some reports have indicated that hyperuricemia leads to increased mortality among hemodialysis patients, but some studies have also shown that the relationship between SUA level and all-cause mortality in hemodialysis patients is reflected by a J-shaped curve [6,7]. In addition, many reports have found that lower uric acid levels are associated with a higher risk of death among hemodialysis patients [8–12]. Therefore, there is still much controversy about the relationship between SUA levels and all-cause or CV mortality in hemodialysis patients. Specific data on the relationship between SUA and outcomes among Chinese patients undergoing hemodialysis are lacking.

In this retrospective cohort study, we assessed the relationship between SUA levels and all-cause and CV mortality and explored the correlation between SUA levels and clinical variables to further reveal the potential mechanism.

## Material and methods

### Inclusion and exclusion criteria

A total of 210 patients who underwent hemodialysis at the Blood Purification Center of the Third Affiliated Hospital of Sun Yat-Sen University from 1 January 2011 to 30 December 2015 were enrolled in the cohort. All patients received hemodialysis three times a week for four hours each time and HDF once every two weeks. The exclusion criteria were as follows: (1) patients whose maintenance hemodialysis time was less than 3 months; (2) cancer patients; (3) patients with incomplete key baseline data, including SUA, complications, body mass index (BMI), etc.; and (4) patients undergoing peritoneal dialysis or kidney transplantation. This study was approved by the Ethics Committee of the Third Affiliated Hospital of Sun Yat-Sen University (approval No.: [2016] 2-56). Patients were stratified into the following groups according to the concentration of SUA: group 1, <420 µmol/L (< 20th percentile); group 2, 420–644 µmol/L (20th to 80th percentile); group 3, >644 µmol/L (>80th percentile).

### Data collection

Baseline demographic and clinical data were collected, include age, sex, primary disease, BMI, diabetes mellitus, hypertension, CV and cerebrovascular diseases, systolic and diastolic blood pressure, urea nitrogen clearance rate (Kt/V), hemoglobin (HGB), serum creatinine (Scr), serum albumin (Alb), SUA, the geriatric nutritional risk index (GNRI), total cholesterol (TC), triglycerides (TG), low density lipoprotein cholesterol (LDL-C), high-sensitivity C-reactive protein (hs-CRP), serum calcium (Ca), phosphorus (P), and parathyroid hormone (PTH) were recorded. The SUA value of all patients is got before long-term hemodialysis, and Uricase-PAP method was used to measure SUA in laboratory. The GNRI was assessed from individual serum albumin concentration and body weight, as reported by Yamada et al. [13], using the following equation: GNRI= (14.89 × albumin [g/dL]) + (41.7 × [body weight/ideal body weight]). Ideal weight (kg) = height (cm) − 105. The length of follow-up, patient outcomes, causes of death, and other information were also recorded. Carotid intima-media thickness (cIMT) and carotid plaques were assessed by a MicroMaxx Ultrasound system paired with a 5–10 MHz multifrequency high-resolution linear transducer (SonoSite, Bothell, WA, USA). The main outcome was all-cause mortality, and the secondary outcome was CV mortality. CV death included heart failure, myocardial infarction, fatal arrhythmia, sudden death, cerebral hemorrhage, and cerebral infarction. Renal transplant patients, peritoneal dialysis patients, and patients with missed visits were recorded as being censored.

### Statistical analysis

The data were processed using SPSS 20.0 software (IBM Corp, USA). Continuous variables conforming to a normal distribution were expressed by the mean value plus the standard deviation, and to nonnormal distribution were expressed as M (1/4, 3/4). Categorical variables were presented as numbers with percentages. Student’s *t* test was used for the comparison of continuous variables with homogeneity of variance; otherwise, the Wilcoxon rank sum test was used. Fisher’s exact probability test or *χ*^2^ test was used to compare the categorical variables. The Pearson correlation coefficient was used to assess the correlation between single variables and SUA levels, and partial correlation was used to correct for age and sex. The survival rate was calculated by the Kaplan–Meier method, and the survival curve was compared with the log-rank test. The survival receiver operating characteristic (ROC) curve was used for area under curve (AUC) analysis to predict mortality at a specific time based on uric acid levels. A Cox proportional hazard model was used to assess the association between SUA level and all-cause mortality and CV mortality in maintenance hemodialysis patients and was expressed as the hazard ratio (HR) and 95% confidence interval (95% CI). The following variables were adjusted in the model: age, sex, hypertension, diabetes, and CV diseases. *p* < .05 was considered to be statistically significant.

## Results

### Baseline demographic, clinical, and laboratory characteristics

The baseline demographic and clinical data are shown in Table 1. A total of 210 patients were enrolled in the study, with an average age of 56.6 ± 16.6 years; 59.5% were male, 63.8% had hypertension, 33.3% had diabetes, and 26.2% had CV or cerebrovascular diseases. The average baseline Scr was 972.8 ± 386.5 µmol/L, and the average baseline SUA was 532.5 ± 137.4 µmol/L. The results showed that the patients in the lowest quintile group were older and had a higher prevalence of diabetes mellitus than the patients in the highest quintile group (*p* < .05). In addition, compared with the highest quintile group patients, serum ALB, P, Scr, and GNRI were lower in the lowest quintile group patients (*p* < .05). Hs-CRP was significantly elevated in the lowest quintile group (*p* < .05). Mean intima-media thickness was significantly higher in the lowest quintile group than in the other groups, as was the prevalence of carotid plaques. There were no significant differences in blood pressure, BMI or lipid levels among the three groups. Moreover, there was no significant difference in medication such as aspirin, losartan, atrovastatin that may affect the level of uric acid among the three groups.

**Table 1.** Baseline data and prognosis of MHD patients grouped by quintile of serum uric acid (SUA) level (*n* = 210).

Variable	Overall (*n* = 210)	Q1 (*n* = 42)	Q2 (*n* = 126)	Q3 (*n* = 42)
Age (year)	56.6 ± 16.6	65.2 ± 14.6	54.4 ± 16.8^a^	54.7 ± 15.3^a^
Female/Male	85/125	15/27	58/68	12/30
Hypertension [cases (%)]	134 (63.8%)	27 (64.3%)	81 (64.3%)	26 (61.9%)
Diabetes [cases (%)]	70 (33.3%)	21 (50.0%)	40 (31.7%)	9 (21.4%)^a^
CV disease[cases (%)]	55 (26.2%)	17 (40.5%)	31 (24.6%)	7 (16.7%)^a^
SBP (mmHg)	148 ± 27	145 ± 26	149 ± 28	150 ± 25
DBP (mmHg)	78 ± 16	75 ± 18	78 ± 16	79 ± 15
Kt/v	1.47 ± 0.68	1.39 ± 0.46	1.52 ± 0.74	1.46 ± 0.64
BMI (kg/m^2^)	22.6 ± 3.9	23.1 ± 4.2	22.8 ± 3.9	21.8 ± 3.2
HGB (g/L)	100.0 ± 23.1	94.2 ± 19.5	101.2 ± 24.3^a^	102.2 ± 22.3
ALB (g/L)	37.2 ± 4.6	35.5 ± 5.3	37.3 ± 4.1^a^	38.5 ± 4.7^a^
Ca (mmol/L)	2.20 ± 0.27	2.15 ± 0.27	2.21 ± 0.26	2.17 ± 0.30
P (mmol/L)	1.92 ± 0.62	1.64 ± 0.50	1.91 ± 0.63^a^	2.20 ± 0.59^b^
PTH (pmol/L）	357.4 (183.9,667.6)	305.5 (153.2,533.7)	379.1 (194.7,695.5)	365.4 (149.6,739.3)
hs-CRP (mg/L)	3.1 (1.2,11.2)	9.0 (2.2,31.3)	2.6 (1.1,8.0) ^b^	2.8 (0.9,7.6)^b^
Scr (μmol/L)	972.8 ± 386.5	742.4 ± 275.4	981.7 ± 370.8^b^	1176.5 ± 411.6^b^
UA (μmol/L)	532.5 ± 137.4	342.6 ± 64.5	532.1 ± 60.7^b^	723.5 ± 81.1^b^
TC (mmol/L)	4.19 ± 1.13	4.06 ± 1.26	4.19 ± 1.14	4.29 ± 1.01
TG (mmol/L)	1.39 ± 0.63	1.34 ± 0.62	1.38 ± 0.62	1.47 ± 0.69
LDL (mmol/L)	2.45 ± 0.92	2.37 ± 0.87	2.41 ± 0.96	2.63 ± 0.85
GNRI	98.7 ± 8.6	95.4 ± 7.5	99.4 ± 8.6^a^	99.9 ± 9.4^a^
Gout (cases,%)	13 (6.2)	2 (4.8)	8 (6.3)	3 (7.1)
Mean intima-media thickness (mm)	0.8 ± 0.2	0.90 ± 0.30	0.70 ± 0.20^b^	0.70 ± 0.30^b^
Prevalence of carotid plaques (%)	57.6	78.6	51.6^a^	54.8^a^
All-cause death (cases, %)	68 (32.4%)	22 (52.4%)	40 (31.7%)^a^	6 (14.3%)^b^
CV death (cases, %)	34 (16.2%)	12 (28.6%)	20 (15.9%)^a^	2 (4.8%)^b^
Aspirin	73 (34.8%)	18 (42.9%)	42 (33.3%)	13 (31.0%)
Losartan	26 (12.4%)	5 (11.9%)	14 (11.1%)	7 (16.7%)
Amlodipine	57 (27.1%)	13 (31.0%)	31 (24.6%)	13 (31.0%)
Atrovastatin	63 (30.0%)	17 (40.5%)	34 (27.0%)	12 (28.6%)

Except for the labeling, the remaining data are expressed in x ± s; the overall comparison of measurement data is based on one-way ANOVA, and the two comparisons are based on corrected *t*-test; the overall comparison of counting data is based on R × C Table χ2 analysis, and the two comparisons are based on Chi-square segmentation method; compared with Q1 group.

^a^ *p* < .05.

^b^ *p* < .01.

### Survival analysis

**Figure 1.**
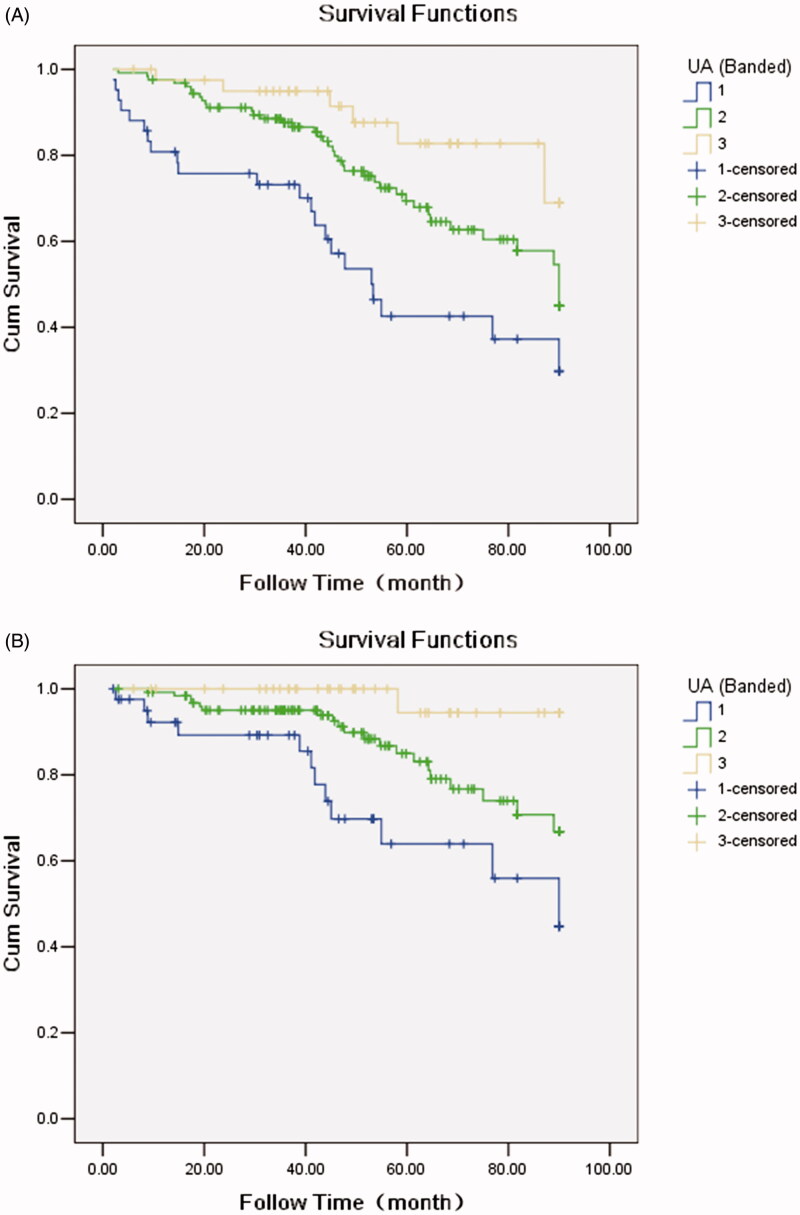
(A) Kaplan–Meier survival curve of MHD patients grouped by quintiles of serum uric acid (SUA) level (all-cause mortality) [Log Rank *χ*^2^=15.61, *p* = .000]. (B) Kaplan–Meier survival curve of MHD patients grouped by quintiles of SUA level (cardiovascular mortality) [Log Rank *χ*^2^=14.28, *p* = .000].

Of the 210 patients enrolled in the cohort, the longest follow-up time was 90 months, and the shortest follow-up time was 2 months. The median follow-up time was 49.8 months. A total of 68 (32.4%) all-cause deaths and 34 (16.2%) CV deaths were recorded during the follow-up period. The causes of death were cardiogenic (22 cases, 32.4%), infection (16 cases, 23.5%), cerebrovascular disease (12 cases, 17.6%), systemic failure (8 cases, 11.8%), hyperkalemia (3 cases, 4.4%), and other causes (7 cases, 10.3%). CV death included heart failure (10 cases, 29.4%), cerebral hemorrhage (10 cases, 29.4%), myocardial infarction (5 cases, 14.7%), sudden death (4 cases, 11.8%), fatal arrhythmia (2 cases, 5.9%), massive cerebral infarction (2 cases), and pulmonary hypertension (1 case, 2.9%). Comparing the prognosis of different SUA groups, it was found that all-cause mortality and CV mortality in the lowest quintile of the SUA group were significantly higher than those in the other two groups (Table 1). The top three causes of death were heart failure (8 cases), infection (7 cases), and cerebral hemorrhage (4 cases). The Kaplan–Meier survival curve showed that with the decrease in SUA, all-cause mortality (log rank*χ*^2^ = 15.61, *p* = .000) and CV mortality (log rank*χ*^2^ = 14.28, *p* = .000) increased (Figure 1(A,B)). Further, survival ROC curve was used to analysis AUC, and the result demonstrated that AUC for all-cause mortality and CVD mortality were 0.635 and 0.866, respectively.

### Effect of SUA level on all-cause and CV mortality

Univariate Cox regression analysis showed that age, hypertension, diabetes mellitus, CV and cerebrovascular diseases, BMI, HGB, ALB, hs-CRP, Scr, and UA (each 100 µmol/L reduction) were associated with all-cause mortality, but further multivariate Cox regression showed that only age, hs-CRP, and Scr were independent risk factors for all-cause mortality. However, in patients who experienced CV death, multivariate regression showed that a history of CV and cerebrovascular disease, albumin level, and uric acid level were independent risk factors for CV death (see Table 2).

**Table 2.** Risk factors for all-cause mortality and cardiovascular mortality in MHD patients (Cox regression analysis).

	Univariate		Multivariate	
Covariate	HR (95% CI)	*p* Value	HR (95% CI)	*p* Value
All-cause mortality				
Age at HD onset (per 1 year)	1.041(1.023–1.059)	.000	1.025(1.006–1.045)	.012
Hypertension	2.481(1.378–4.469)	.002	1.656(0.783–3.506)	.187
DM	2.300(1.423–3.715)	.001	0.970(0.540–1.742)	.918
CV disease	2.230(1.375–3.615)	.001	0.995(0.929–1.066)	.152
BMI	1.065(1.005–1.127)	.033	1.495(0.863–2.590)	.896
HGB	0.989(0.981–0.998)	.013	0.995(0.983–1.007)	.403
ALB	0.921((0.876–0.968)	.001	0.964(0.919–1.012)	.141
hs-CRP	1.006(1.002–1.011)	.003	1.007(1.001–1.012)	.018
Scr	0.998(0.997–0.999)	.000	0.999(0.998–1.000)	.004
UA (per 100 μmol/L)	0.691(0.574–0.833)	.000	0.918(0.746–1.129)	.417
CV mortality				
Age at HD onset (per 1 year)	1.026(1.002–1.050)	.032	0.998(0.972–1.025)	.882
DM	1.915(0.966–3.795)	.063	0.658(0.288–1.507)	.323
CV disease	3.364(1.699–6.661)	.000	3.268(1.209–7.580)	.006
ALB	0.914(0.851–0.981)	.013	0.906(0.828–0.991)	.030
hs-CRP	1.005(0.999–1.012)	.098	1.005(0.996–1.013)	.264
Scr	0.998(0.997–0.999)	.000	0.999(0.997–1.000)	.080
UA (per 100 μmol/L)	0.608(0.465–0.794)	.000	0.693(0.479–0.975)	.036

We further used the Cox proportional hazard model to quantify the relationship between SUA levels and all-cause and CV mortality (Table 3). Based on the continuous linear model, in the unadjusted Cox proportional hazard model, lower SUA was associated with higher all-cause, and CV mortality. Considering that the group with the lowest SUA quintile level was older and had a higher proportion of diabetes mellitus and CV and cerebrovascular diseases, we adjusted for age, sex, carotid plaques, and complications. We found that for every 100 µmol/L increase in SUA level, the HR was 0.792 (95% CI, 0.645–0.972); *p* = .026). The risk of all-cause death in the lowest SUA quintile group was 2.85 times higher than that in the highest SUA quintile group. For every 100 µmol/L increase in SUA, the HR of CV death was 0.683 (95% CI, 0.505–0.924; *p* = .014), suggesting a more significant decrease in CV mortality. Further comparisons between the groups with the lowest SUA quintile level showed that the risk of CV death was nine times higher in the group with the lowest SUA quintile level than in the group with the highest SUA quintile level.

**Table 3.** Hazard ratio of all-cause mortality and CV mortality associated with continuous and quintiles of SUA levels in unadjusted and multivariable-adjusted Cox proportional hazards models.

Variable	Event	Unadjusted	*p* Value	Multivariable adjusted^a^	*p* Value
All-cause mortality^b^	68/210 (32.4%)	0.691 (0.574–0.833)	.000	0.792(0.645–0.972)	.026
Quintile of uric acid level					
Group 1	22/42 (52.4%)	Reference		Reference	
Group 2	40/126 (31.7%)	0.481 (0.286–0.810)	.006	0.719 (0.402–1.287)	.267
Group 3	6/42 (14.3%)	0.210 (0.085–0.519)	.001	0.351 (0.132–0.934)	.036
CV mortality^b^	34/210 (16.2%)	0.608 (0.465–0.794)	.001	0.683 (0.505–0.924)	.014
Quintile of uric acid level					
Group 1	12/42 (28.6%)	Reference		Reference	
Group 2	20/126 (15.9%)	0.432 (0.211–0.884)	.022	0.618 (0.270–1.416)	.256
Group 3	2/42 (4.8%)	0.063 (0.008–0.486)	.008	0.112 (0.014–0.925)	.042

^a^ Adjusted for the factors of age, sex, carotid plaques, complications (hypertension, diabetes mellitus, and CV disease).

^b^ Continuous per 100 μmol/L increase in uric acid.

**Table 4.** Analysis of correlation between SUA level and clinical variables.

Variable	Unadjusted *r*	*p* Value	*r* adjusted for age, sex	*p* Value
ALB	0.179	.011	0.135	.047
P	0.275	.000	0.249	.000
hs-CRP	−0.203	.003	−0.202	.003
Scr	0.362	.000	0.305	.000
GNRI	0.174	.012	0.156	.024

*r* = correlation coefficient.

### The correlation between SUA level and clinical variables

The Pearson correlation coefficient was used to explore the correlation between SUA and clinical variables (Table 4). The SUA level was positively correlated with nutritional factors such as ALB (*r* = 0.135, *p* < .05), Scr (*r* = 0.305, *p* < .001), P (*r* = 0.249, *p* < .001), and GNRI (*r* = 0.156, *p* < .05). In addition, SUA was negatively correlated with hs-CRP (*r* = −0.202, *p* < .01).

## Discussion

Our study demonstrated that SUA levels were an independent risk factor for CV mortality, but not all-cause mortality, among hemodialysis patients. After adjusting for age, sex, and complications, the risk of all-cause and CV death in the low SUA quintile was significantly higher. Further correlation analysis revealed that SUA levels were correlated with nutritional and inflammatory status.

Increasing evidence suggests that higher SUA levels may be a risk factor or biomarker for kidney and CV outcomes in the general population. In humans, approximately two-thirds of the uric acid produced needs to be cleared by the kidney. Therefore, in CKD patients, the phenomenon of increased SUA due to impaired renal function is very common. However, for MHD patients, the correlation between SUA and prognosis remains a controversial topic. Previous studies have shown that there is a J-shaped curve between SUA and the survival rate of hemodialysis patients [6,7]. However, the sample size of the study that was used to reach this conclusion was less than 300, and the SUA level was collected before dialysis for once. Subsequently, a series of larger studies [8,11,12] found that patients with higher uric acid levels had a lower risk of all-cause and CV death. A prospective observational study of 261 MHD patients also showed that for each 1 mg/dL increase in baseline SUA, the risk ratio of all-cause and CV death decreased by 45% [14]. Consistent with the above research, we have reached a similar conclusion.

The mechanism of the association between SUA level and increased mortality in hemodialysis patients has not been fully elucidated. Currently, two possible underlying mechanisms have been considered. First, SUA is considered an indicator of the nutritional status of dialysis patients, and nutritional status is an important factor affecting the long-term survival of hemodialysis patients. Current studies have found that high uric acid levels are positively correlated with nutritional status indicators such as BMI, nPCR, serum albumin, phosphorus, and creatinine levels. The increase in serum albumin has been confirmed to be associated with a reduction in mortality among hemodialysis patients [15]. These findings were also supported by our study. We used GNRI, a simplified indicator of nutritional status, to assess the nutritional status of MHD patients. The GNRI has been applied for the assessment of protein energy wasting (PEW) and could offer a strong predictor of mortality in hemodialysis patients [13,16]. In our study, patients with the lowest quintile array of uric acid had lower GNRI than other patients. Correlation analysis showed that the level of uric acid was closely related to GNRI. In addition, current studies also suggest that uric acid is a powerful oxygen free radical scavenger with antioxidant and anti-inflammatory effects [17,18]. Increasing SUA levels in volunteers has been shown to prevent exercise-induced increases in plasma isoprostaglandins [19]. It was also found that increasing uric acid levels in hypercholesterolemic mice prevented oxidative inactivation of superoxide dismutase containing copper/zinc [20]. In dialysis patients, total antioxidant capacity was found to be associated with higher SUA levels [21]. We also found that the level of hs-CRP in patients with the lowest uric acid quintile increased significantly, suggesting that the inflammatory state in patients with low uric acid was more significant.

Current studies have suggested that nontraditional CV risk factors, such as anemia, nutritional status, oxidative stress, and microinflammation, play a more important role in promoting CV disease in hemodialysis patients [22,23]. Our study suggested that hypouricemia is closely associated with MIA syndrome (malnutrition–inflammation–atherosclerosis) [24,25]. The patients in the hypouricemia group who had a high prevalence of carotid plaques and increased carotid intima-media thickness had lower albumin levels and higher hs-CRP levels. Two studies clearly demonstrated the close association between MIA syndrome and high mortality among HD patients [26,27]. We speculated that malnutrition can reduce the volume of cardiac myocytes and the content of myofibrils, cause an increase in fibrinogen, induce oxidative stress and vascular inflammation, and promote atherosclerosis. In addition, some studies have confirmed the role of uric acid in improving vascular endothelial dysfunction caused by uremic toxin. Endothelial dysfunction caused by related toxins in the uremic environment, such as indole sulfate, causes endothelial dysfunction by reducing the production of nitric oxide and increasing oxidative stress, leading to adverse effects in the context of CV diseases [28,29]. High SUA levels can block toxin action in the Akt-eNOS-NO pathway, increase NO levels, and reduce the production of reactive oxygen species in mitochondria, thereby improving the endothelial cell function induced by indolephenol sulfate [30]. Therefore, among hemodialysis patients, high SUA levels may be a compensatory mechanism to counteract oxidative damage and vascular toxicity from uremic toxins such as indolephenol sulfate.

This study has some limitations. First, due to that our study was a single-center study with a limited number of patients enrolled, it was difficult to adapt this result to the general population. Second, because this study was designed as a retrospective cohort study, some patients without SUA data or with other incomplete data were excluded, resulting in selection bias to a certain extent. In addition, there may be a problem related to not including patients with high uric acid levels but who have died. Third, this study used baseline SUA values rather than mean uric acid values during follow-up to explore the relationship with mortality, which is also a limitation. Finally, this study did not fully include the effect of drugs on uric acid , which would also lead to some deviations in the results of the study.

In summary, our study showed that in MHD patients, a decrease in baseline SUA levels was closely related to an increased risk of all-cause and CV death. However, further multicenter prospective trials are needed to confirm these findings, and further basic studies are needed to clarify the exact mechanism behind the findings.
